# Exposure to Δ9-tetrahydrocannabinol during rat pregnancy leads to impaired cardiac dysfunction in postnatal life

**DOI:** 10.1038/s41390-021-01511-9

**Published:** 2021-04-20

**Authors:** Kendrick Lee, Steven R. Laviolette, Daniel B. Hardy

**Affiliations:** 1 Department of Physiology and Pharmacology, Western University, London, ON Canada; 2 Department of Anatomy and Cell Biology, Western University, London, ON Canada; 3 Departments of Obstetrics and Gynecology, Children’s Health Research Institute, Lawson, Health Research Institute, Western University, London, ON Canada

## Abstract

**Background:**

Cannabis use in pregnancy leads to fetal growth restriction (FGR), but the long-term effects on cardiac function in the offspring are unknown, despite the fact that fetal growth deficits are associated with an increased risk of developing postnatal cardiovascular disease. We hypothesize that maternal exposure to Δ9-tetrahydrocannabinol (Δ9-THC) during pregnancy will impair fetal development, leading to cardiac dysfunction in the offspring.

**Methods:**

Pregnant Wistar rats were randomly selected and administered 3 mg/kg of Δ9-THC or saline as a vehicle daily via intraperitoneal injection from gestational days 6 to 22, followed by echocardiogram analysis of cardiac function on offspring at postnatal days 1 and 21. Heart tissue was harvested from the offspring at 3 weeks for molecular analysis of cardiac remodelling.

**Results:**

Exposure to Δ9-THC during pregnancy led to FGR with a significant decrease in heart-to-body weight ratios at birth. By 3 weeks, pups exhibited catch-up growth associated with significantly greater left ventricle anterior wall thickness with a decrease in cardiac output. Moreover, these Δ9-THC-exposed offsprings exhibited increased expression of collagen I and III, decreased matrix metallopeptidase-2 expression, and increased inactivation of glycogen synthase kinase-3β, all associated with cardiac remodelling.

**Conclusions:**

Collectively, these data suggest that Δ9-THC-exposed FGR offspring undergo postnatal catch-up growth concomitant with cardiac remodelling and impaired cardiac function early in life.

**Impact:**

- To date, the long-term effects of perinatal Δ9-THC (the main psychoactive component) exposure on the cardiac function in the offspring remain unknown.
- We demonstrated, for the first time, that exposure to Δ9-THC alone during rat pregnancy results in significantly smaller hearts relative to body weight.
- These Δ9-THC-exposed offsprings exhibited postnatal catch-up growth concomitant with cardiac remodelling and impaired cardiac function.
- Given the increased popularity of *cannabis* use in pregnancy along with rising Δ9-THC concentrations, this study, for the first time, identifies the risk of perinatal Δ9-THC exposure on early postnatal cardiovascular health.

## Introduction

Cannabis is the most consumed illicit drug in the world with ~140 million users worldwide.^1^ Among pregnant women in North America, recent studies report that up to 7% use cannabis during pregnancy and ~5% consume it while nursing.^2^ Moreover, these numbers are expected to rise with the legalization of cannabis in Canada and many parts of the United States.^3^ Many use cannabis given the common perception that it mitigates anxiety, depression, and nausea while posing no risk to the baby.^4–6^ This is concerning given that there are currently three systemic reviews that suggest cannabis consumption during pregnancy leads to low-birth-weight outcomes.^7–9^ However, these studies are confounded by socioeconomic status (SES) and the fact that women tend to co-medicate (i.e., tobacco) during pregnancy.^10^ To date, we and others have demonstrated that exposure (oral, intraperitoneal (i.p.), intravenous (i.v.), or inhalation) to Δ9-tetrahydrocannabinol (Δ9-THC), the main psychoactive component of cannabis, in pregnancy can lead to placental insufficiency and fetal growth restriction in the rat;^11–16^ however, the long-term cardiometabolic effects are unknown. This is of great interest considering that low birth weight offspring is associated with long-term cardiovascular disease.^17^

Δ9-THC natively interacts with the endocannabinoid system, which is composed of two receptors, cannabinoid receptor type 1 and 2 (CB1R and CB2R). In the central nervous system (CNS), the endocannabinoid system mediates appetite, mood, pain, and memory.^18^ Although traditionally only recognized in the CNS, more recently CB1R and CB2R have been localized in peripheral tissues such as the liver, adipose, pancreatic, cardiac, placental, and immune tissue, suggesting that cannabinoids may exert its effects outside the CNS.^19–28^ Δ9-THC can also directly exert its effect via the endocannabinoid system on fetal tissues as it has been found to cross the human placenta and can concentrate 2–5 times higher in fetal tissues compared to that in maternal tissues.^29,30^ In addition, due to selective breeding of cannabis strains, the concentration of Δ9-THC has increased from 4 to 12% in the past two decades.^31^ This is concerning as Δ9-THC could exert direct effects on fetal development. For example, in isolated neonatal cardiomyocytes, CB1R agonists can impair cardiomyocyte size during development.^28^ Conversely, CB1R antagonists have been shown to prevent cell death in embryonic cardiomyocytes.^32^ Collectively, this suggests that in addition to its proposed role to reduce placental efficiency and fetal development, exposure to Δ9-THC could also have direct detrimental effects on the developing heart. Therefore, in this present study, we investigated if low-birth-weight offspring exposed to Δ9-THC *in utero* exhibited defects in functional cardiac outcomes in postnatal life.

## Materials and methods

### Δ9-THC animal model

All animal procedures were conducted in accordance with the guidelines and standards of the Canadian Council on Animal Care. Animal Use Protocol (AUP #2019-126) was approved and post-approval monitoring was conducted by the Western University Animal Care Committee. All investigators understood and followed the ethical principles outlined by Grundy,^33^ and the study design was informed by ARRIVE (Animal Research: Reporting of In Vivo Experiments) guidelines.^34^ Time-pregnant Wistar rat dams were purchased from Charles River (La Salle) and were maintained at 22 °C on a 12:12-h light–dark cycle with access to food and water *ad libitum* throughout the experimental procedure. Dams arrived at the animal facility at gestational day (GD) 3 and were left to acclimatize for 3 days. From there, animals were randomly assigned to a treatment group and administered 3 mg/kg of Δ9-THC (*N* = 8) or saline as a vehicle (*N* = 8) daily via i.p. injection from GD 6 to 22 (birth), as we have previously performed.^11^ This dose was selected as it results in a plasma concentration range in rodents (8.6–12.4 ng/mL) similar to that of human (13–63 ng/mL) cannabis smokers (using 6% Δ9-THC).^35–37^ We avoided the oral route considering it has poorer bioavailability and slower adsorption with food along with the fact that edibles are the least popular route of *cannabis* consumption in pregnant women.^38,39^ *In utero* exposure to Δ9-THC earlier than GD 6 has been shown to result in spontaneous abortions in rats.^38^ Previously, we and others have demonstrated that this dose and route of Δ9-THC administration does not alter maternal outcomes or lead to fetal demise.^11,13,35,40,41^ For both treatment groups, pups were culled to 8 pups/mothers (4 males, 4 females) to ensure standardized postnatal nutrition. At birth, male and female hearts were harvested and weighed from culled pups. The remaining pups were studied longitudinally by echocardiography (echo) at postnatal days 1 (PND1) and 21 (PND21). For the purposes of this study, male offsprings were exclusively selected to avoid confounding effects presented by the female estrus cycle and to reduce costs associated with echoes. After the echoes, pups were sacrificed using an overdose of pentobarbital (100 mg/kg) i.p. for heart tissue collection and flash-frozen in liquid N_2_ for molecular analysis.

### Echocardiographic assessment of cardiac function

The Vevo2100 Ultrasound Imaging System was employed to obtain two-dimensional echocardiographic footage in parasternal short axial (M-mode) and long axial (B-mode) views using a 40 MHz linear transducer. Animals were sedated using isoflurane throughout the duration of the echoes . Heart rate was measured using electrode probes on the extremities and body temperature was monitored using a rectal probe. Real-time images obtained in the short axial view were used to measure left ventricular interior diameter (LVID) and posterior (LVPW) and anterior wall thickness (LVAW) at systolic and diastolic contraction. Using M-mode and B-mode, estimates were made for stroke volume, ejection fraction, fractional shortening, and cardiac output.

### RNA extractions and real-time RT-qPCR

Total RNA was extracted from whole-heart rat tissue in TRIzol reagent (Invitrogen, Carlsbad, CA) for 30 s and then subsequently homogenized using a homogenizer. Chloroform (Sigma-Aldrich, St. Louis, MO) was added, shaken, and then centrifuged at 12,500 r.p.m. at 4 °C for 15 min. Approximately 500 mL of the supernatant was taken and mixed with equal volumes of isopropyl alcohol and chilled at −20 °C for 20 min. The solution was then centrifuged for 15 min and the supernatant was decanted and the pellet of RNA was retrieved and washed with ethanol. After washing, the pellet was dissolved in DEPC (diethylpyrocarbonate)-treated water and quantified using Nanodrop 2000 (Thermo Fisher Scientific, Waltham, MA) and diluted to 2 μg of RNA. Using Superscript II Reverse Transcriptase Kit (Invitrogen), 2 μg of RNA was reverse-transcribed to make complementary DNA (cDNA). cDNA was diluted 1:40. Primer sets for *Collagen* 1 (NM_053304.1: forward 5′-GTACATCAGCCCAAACCCCA-3′; reverse 5′-TCGCTTCCATACTCGAACTGG-3′) *Collagen* 3 (NM_032085.1: forward 5′-GAAAGGTGAAATGGGTCCAGC-3′; reverse 5′-CTTTGCTCCATTCTTGCCCG-3′), *β-actin* (NM_031144: forward 5′-CACAGCTGAGAGGGAAAT-3′; reverse 5′-TCAGCAATGCCTGGGTAC-3′), and *GAPDH* (glyceraldehyde 3-phosphate dehydrogenase) (NM_017008.4: forward 5′-GGATACTGAGAGCAAGAGAGAGG-3′; reverse 5′-TCCTGTTGTTATGGGGTCTGG-3′) in the rat were designed using the National Center for Biotechnology Information and Ensemble genome browsers, followed by generation via Invitrogen Custom DNA Oligos. SsoFast Eva green supermix (Bio-Rad) and Bio-Rad CFX384 Real-Time System were used with cyclic conditions set at 95 °C for 10 min, followed by 43 cycles of 95 °C for 15 s and 60 °C for 30 s and 72 °C for 30 s. Relative messenger RNA (mRNA) abundance obtained for all target genes of interest was normalized to geometric means of β-actin and GAPDH. β-Actin and GAPDH were determined to be suitable housekeeping genes by using both the comparative delta Ct method and algorithms from geNorm, Normfinder, and BestKeeper.^42,43^ Primer efficiency was determined to be equal for all primer sets, and ΔCt values for each primer were calibrated to experimental samples with the lowest transcript abundance (highest Ct value). Relative transcript abundance was then calculated for each primer set as determined by the formula 2ΔΔCt, where ΔΔCt was the normalized value.

### Protein extraction and Western blot

Total protein from whole hearts was extracted by homogenization in a RIPA buffer solution (50 mM Tris-HCl, pH 7.4, 150 mM NaCl, 1 mM EDTA, 1% Nonidet P40, and 0.25% C_24_H_39_NaO_4_, with protease inhibitor cocktail (Roche, Basel, Switzerland)) with phosphatase inhibitors (40 mM Na_3_VO_4_, 40 mM Na-pyrophosphate 20 mM NaF, and 200 mM β-glycerophosphate disodium salt hydrate). Heart cells were further lysed by sonicating the solution for five, 1-s pulses at 30% amplitude and then subsequently mixed using a rotator for 10 min at 4 °C. The solution was then centrifuged for 15 min at 4 °C. The supernatant was collected and aliquoted as total protein and then quantified using a Lowry Protein Assay Kit (Bio-Rad, Hercules, CA). Once quantified, loading mixes were prepared by diluting proteins to 20 μg/well and mixed with NuPAGE Reducing Agent (10×) (Invitrogen), NuPAGE LDS Sample Buffer (4×) (Invitrogen), and deionized water. Protein samples were heated at 70 °C for 10 min to denature the proteins and were separated by gel electrophoresis using a gradient gel (Novex, Thermo Fisher Scientific). Gels were then transferred using polyvinylidene difluoride membranes (Millipore, Billerica, MA). Membranes were flooded with Ponceau S and shaken for 1 min and then imaged for total protein abundance using a ChemiDoc Imager (Bio-Rad). Membranes were blocked in 5% non-fat milk or 5% bovine serum albumin (in 1× TBST). Membranes were then probed overnight with primary antibodies (in blocking agent): collagen I (1:1000 dilution, Abcam, #ab34710, Cambridge, MA), collagen IIIA1 (1:500 dilution, Santa Cruz, sc-271249, Santa Cruz, CA), matrix metalloproteinase-2 (MMP-2) (1:1000 dilution, Cell Signalling Technologies, #87809, Beverly, MA), phosphorylated glycogen synthase kinase-3β (GSK-3β) [serine 9A] (1:1000 dilution, Cell Signalling Technologies, #9336), and total GSK-3β (1:1000 dilution, Cell Signalling Technologies, #12456). Horse anti-mouse (1:10,000 dilution, Cell Signalling Technologies, #7076P2) and goat anti-rabbit (1:10,000 dilution, Cell Signalling Technologies, #7074P2) secondary antibodies were diluted in the blocking solution and rotated at room temperature for 1 h. Immunoreactive bands were detected using Super Signal West Dura Chemiluminescent Substrate (Thermo Fisher Scientific) and imaged using a ChemiDoc Imager (Bio-Rad). Relative band density was normalized to total protein using 0.1% Ponceau and quantified using the Image Lab software, as we have previously published.^44^

### Statistical analysis

To avoid litter bias, offspring were taken from separate litters (i.e., *N* = 1 represents pups from a single dam) to achieve *N* = 7–8/group from each of the time points (birth and PND21). This sample size of 7–8 offspring per sex per age group per treatment was chosen based on achieving a statistically significant difference with an expected standard deviation of 15% or less, based on our previous studies.^45,46^ All analyses were completed with GraphPad 8 Prism software using a Student’s unpaired *t-*test. Values depicted are mean ± SEM and considered significant if *p* < 0.05. Grubb’s test was employed to determine outliers.

## Results

### *In utero* exposure to Δ9-THC leads to fetal growth deficits and postnatal catch-up growth

**Fig. 1:**
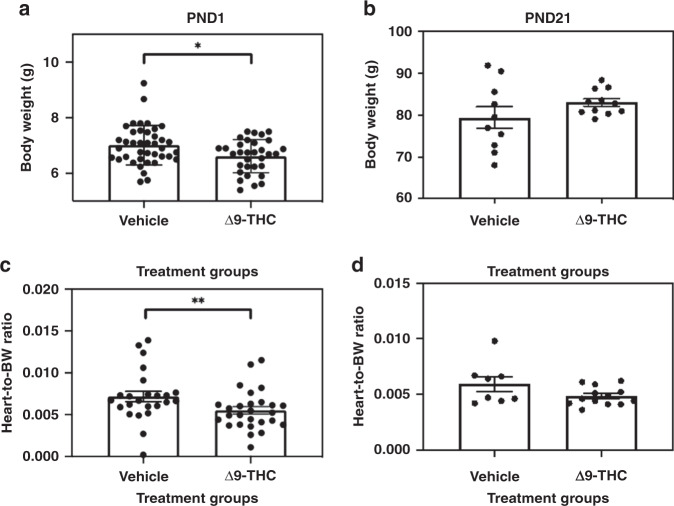
Gestational exposure to Δ9-THC leads to decreased heart weights at birth followed by postnatal catch-up growth at 3 weeks. **a**, **b** Body weights, **c**, **d** heart-to-body weight ratios. All values are expressed as means ± SEM, an average of 4 pups/dam from *N* = 7–8 dams/group (i.e., *N* = 1 represents pups from a single dam). Significant differences between groups were determined using Student’s unpaired *t* test (*p < 0.05, **p < 0.001).

To determine if Δ9-THC exposure *in utero* impedes fetal growth and compromises heart development, offspring that were exposed to either vehicle or 3 mg/kg/day of Δ9-THC i.p. from GD 6 to parturition were measured for body weights and heart-to-body weight ratios at birth. It should be noted that in this same cohort of vehicle and Δ9-THC offspring, we have published that exposure to Δ9-THC in pregnancy did not lead to changes in maternal food intake, maternal weight gain, litter size or gestational length.^11^ At birth, male and female offspring exhibited significantly decreased body weights (Fig. 1a, *p* < 0.05) and heart-to-body weight ratios (Fig. 1c, *p* < 0.01). The range in birth weight for our offspring was 5.4–9.3 g and the 10th percentile birth weight was 5.9 g. Male and female offspring body weights or heart-to-body weight ratios were not different from each other in either experimental group. At 3 weeks of age, these measurements were also followed up to assess postnatal catch-up growth. Three-week-old male Δ9-THC-exposed offspring caught up in growth relative to the vehicle control group (Fig. 1b). Heart sizes relative to body weights also recovered by 3 weeks (Fig. 1d).

### *In utero* exposure to Δ9-THC leads to increased heart rate concomitant with decreased stroke volume at birth

To assess the effects of Δ9-THC on the cardiac function of the offspring, echo measurements were taken in postnatal life. At birth, there was a significant 25% increase in heart rate followed by a compensatory decrease in stroke volume, which resulted in no significant differences in cardiac output (*p* < 0.01, Table 1). All other hemodynamic parameters (i.e., fractional shortening, ejection fraction, and cardiac output) and ventricular wall thicknesses (i.e., LVAW and LVPW) were normal relative to vehicle controls.

**Table 1:** Cardiac functions of offspring at birth exposed to 9-THC throughout gestation.

Parameter	Vehicle (*N* = 8)	Δ9-THC (*N* = 8)	*P* value
Heart rate (b.p.m.)	223.7 ± 15.69	290.1 ± 3.690**	0.0062
Stroke volume (μL)	14.70 ± 1.041	8.933 ± 0.3027**	0.0018
Cardiac output (mL/min)	3.325 ± 0.4601	2.588 ± 0.06122	0.1636
Ejection fraction (%)	67.09 ± 5.274	65.46 ± 1.894	0.7800
Fractional shortening (%)	31.22 ± 4.660	25.09 ± 2.619	0.2955
LVAW; systole (mm)	0.8075 ± 0.09196	0.8425 ± 0.05921	0.7598
LVAW; diastole (mm)	0.4975 ± 0.07146	0.4850 ± 0.04481	0.8870
LVPW; systole (mm)	0.9450 ± 0.1031	1.075 ± 0.1270	0.4571
LVPW; diastole (mm)	0.6750 ± 0.1017	0.7700 ± 0.09065	0.5117
LVID; systole (mm)	1.493\ ± 0.2001	1.378 ± 0.06156	0.6026
LVID; diastole (mm)	2.538 ± 0.1045	2.395 ± 0.1008	0.3643

All values are expressed as means ± SEM, *N* = 8 pups/group (each pup was taken from a different dam’s litter). Student’s unpaired *t* test was used for analysis.

**Significant differences at *p* < 0.01 between vehicle and Δ9-THC. LVAW, left ventricular anterior wall; LVID, left ventricular interior diameter; LVPW, left ventricular posterior wall. All animals were measured at postnatal day 1.

### Three-week-old offspring exposed to Δ9-THC *in utero* exhibit adverse myocardial structure and function

**Fig. 2:**
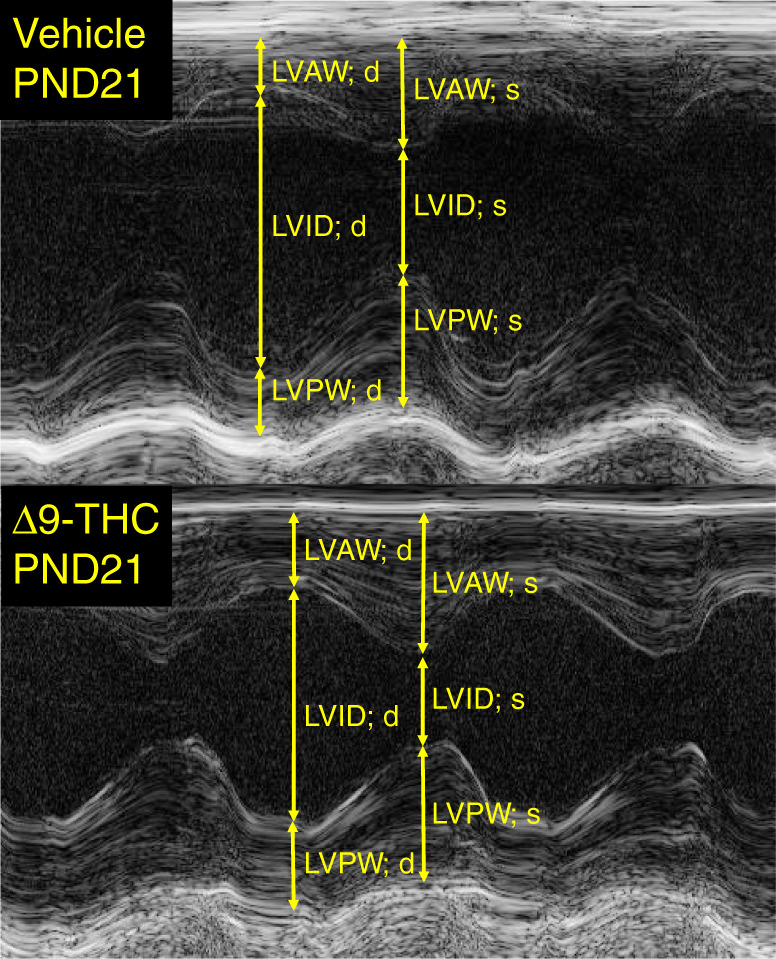
Representative echocardiogram from 3-week vehicle and 3-week-old rat offspring exposed to Δ9-THC. All animals were measured at postnatal day 21. LVAW left ventricular anterior wall, LVID left ventricular interior diameter, LVPW left ventricular posterior wall, d diastole, s systole.

Given that our male 3-week-old offspring exhibited postnatal catch-up growth, which is associated with an increased risk of cardiovascular disease,^47,48^ we further measured cardiac functional outcomes. Echo analysis (Fig. 2) revealed that 3-week-old offspring exposed to Δ9-THC exhibited morphological changes such as thicker anterior left ventricular wall thickness, most noticeable during systolic contraction (*p* < 0.05, Table 2). The LVPW; diastole, although nonsignificant, was also trending towards increased thickness. There were no other significant changes in other functional parameters and the diameter of the left ventricular chamber at systole and diastole. However, while 3-week-old offspring exposed to Δ9-THC exhibited no changes in heart rate, there was a significant 20% decrease in stroke volume (*p* < 0.05, Table 2). Moreover, this culminated in a significant 20% decrease in cardiac output (*p* < 0.05, Table 2).

**Table 2:** Cardiac function of offspring at 3 weeks exposed to 9-THC throughout gestation.

Parameter	Vehicle (*N* = 8)	Δ9-THC (*N* = 8)	*P* value
Heart rate (b.p.m.)	418.6 ± 9.136	419.0 ± 7.415	0.9710
Stroke volume (μL)	149.3 ± 5.720	122.3 ± 10.36*	0.0387
Cardiac output (mL/min)	62.23 ± 1.779	50.02 ± 4.476*	0.0235
Ejection fraction (%)	82.42 ± 2.376	80.37 ± 1.728	0.4974
Fractional shortening (%)	43.57 ± 3.927	36.03 ± 5.864	0.3033
LVAW; systole (mm)	2.160 ± 0.08422	2.564 ± 0.1590*	0.0417
LVAW; diastole (mm)	1.386 ± 0.08187	1.583 ± 0.1117	0.1768
LVPW; systole (mm)	2.088 ± 0.3024	2.333 ± 0.2803	0.5625
LVPW; diastole (mm)	1.528 ± 0.1429	1.949 ± 0.1952	0.1038
LVID; systole (mm)	2.593 ± 0.1284	2.393 ± 0.1068	0.2513
LVID; diastole (mm)	5.201 ± 0.06238	5.006 ± 0.1441	0.2357

All values are expressed as means ± SEM, *N* = 8 pups/group (each pup was taken from a different dam’s litter). Student’s unpaired *t* test was used for analysis.

*Significant differences at *p* < 0.05 between vehicle and Δ9-THC. LVAW, left ventricular anterior wall; LVID, left ventricular interior diameter; LVPW, left ventricular posterior wall. All animals were males measured at 3 weeks of age.

### Three-week-old offspring exposed to Δ9-THC *in utero* exhibit increased markers of cardiac remodelling along with greater cardiac collagen content

**Fig. 3:**
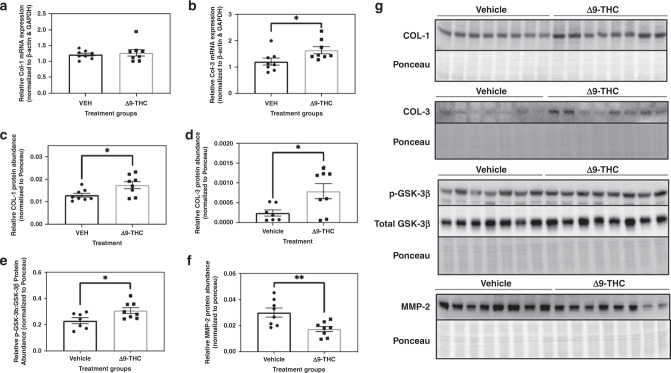
Maternal exposure to 3 mg/kg Δ9-THC i.p. daily from gestational days 6 to 22 results in increased expression of cardiac remodelling markers associated with hypertrophy and collagen deposition. Transcript abundance of **a** collagen I and **b** collagen III. Protein abundance of **c** collagen 1 and **d** collagen III **e** Phosphorylated GSK-3β-to-total GSK-3β ratio and **f** MMP-2. **g** Representative Western blot displaying all the cardiac markers with associated Ponceau staining. All protein levels were expressed as means normalized to total protein (using Ponceau staining), ±SEM, *N* = 7–8 offsprings/group (each offspring was taken from a different dam’s litter). Significant differences between groups were determined using Student’s unpaired *t* test (*p < 0.05, **p < 0.01).

Given that exposed offspring exhibited postnatal catch-up growth, which can be associated with increased risk of developing cardiovascular disease and remodelling,^49,50^ we next sought to elucidate the underlying molecular changes previously associated with cardiac hypertrophy (e.g., GSK-3β). We also wanted to examine whether markers for fibrosis (i.e., collagen I and III and GSK-3β) were upregulated since it has been associated with the development of cardiac hypertrophy.^51^ We observed significant increases in steady-state mRNA transcript abundance for collagen III (Fig. 3b; *p* < 0.05) and a modest increase in collagen I (Fig. 3a). We then investigated whether this led to changes in protein expression. We found that Δ9-THC-exposed offspring exhibited increased protein expression of collagen I and III (Fig. 3c, d; *p* < 0.05). This was further supported by a decrease in protein expression of MMP-2, involved in the breakdown of collagen (Fig. 3f; *p* < 0.01). Given the links between elevated collagen and GSK-3β, we next wanted to determine whether there would be an inactivation of GSK-3β, which results in cardiac hypertrophy and fibrosis in rodents when inactivated (phosphorylated at S9A) or knocked out.^52,53^ Interestingly, we saw a significant increase in the ratio of inactivated (phosphorylated) GSK-3β to total GSK-3β in the Δ9-THC-exposed groups (Fig. 3e).

## Discussion

In the current study, we demonstrated that *in utero* exposure to Δ9-THC alone resulted in fetal growth deficits, including smaller hearts at birth. Furthermore, at 3 weeks, Δ9-THC-exposed offspring exhibited postnatal catch-up growth associated with cardiac remodelling and adverse left ventricular function. Due to the recent legalization of marijuana and the fact that cardiovascular disease is the number one cause of death worldwide,^54^ identifying risks that contribute to the increased likelihood of developing cardiovascular disease is of great relevance. We have previously published, utilizing the same model, that this specific route and dose of Δ9-THC leads to symmetrical intrauterine growth restriction (IUGR), which is exhibited by a proportional decrease of organ and birth weights.^11^ This was associated with altered placental vasculature and nutrient transport, which attributed to the fetal growth deficits observed at birth.^11^ It is worth noting that, using the same dams as the present cohort, this specific route and dosage does not alter maternal food intake or weight gain, and does not lead to fetal demise, which removes confounding effects such as maternal malnutrition and/or litter size.^11^

It is well established that insults during *in utero* development can impede fetal development, which can adversely affect cardiac function and increase the likelihood of developing cardiovascular disease later in life.^48,55,56^ With regards to classifying IUGR, it should be noted that, in models of asymmetrical IUGR, there is an increase in heart-to-body weight ratios (suggesting hypertrophy) indicating a “head sparing effect.”^50,57–59^ In contrast, we have previously published that our specific model using 3 mg/kg Δ9-THC induces placental insufficiency and symmetrical IUGR whereby birth weight is proportionally decreased along with all growth parameters (i.e., liver-to-body weight and brain-to-body weight ratios).^11,59^ This is consistent with our observed decrease in neonatal heart weight relative to body weight in this current study. It is noteworthy to consider that placental insufficiency can result in asymmetric FGR whereby the brain and heart (manifested as hypertrophic heart) are spared. On the contrary, our previously published study demonstrated that gestational exposure to Δ9-THC results in symmetrical IUGR, which is often associated with early gestation insults.^11,59^ With respect to why hypertrophic hearts at birth were not observed in our model, it has been demonstrated that in fetal cardiomyocytes, CB1R and CB2R agonists impede cardiomyocyte growth/hypertrophy.^28^ Given 10% of maternal Δ9-THC results in fetal circulation,^29,30^ this suggests that Δ9-THC, via activation of CB1R in the heart, could have a direct effect on cardiac growth. With the loss of the inhibitory effects of Δ9-THC post partum, we postulate that both body and heart weights are able to catch-up in growth by 3 weeks of age. This is of great interest considering that in other models of IUGR, postnatal cardiometabolic deficits are not observed until only *after* postnatal catch-up growth.^60–62^ Ultimately, this raises concern because in humans, fetal growth deficits and a period of exaggerated rapid growth can be compounded to further increase the risk of cardiovascular disease.^48,56^

Along with decreases in heart size, echo analysis indicates that Δ9-THC-exposed animals at birth had significantly increased heart rate, decreased stroke volume, while maintaining relatively stable cardiac output. The observed tachycardia at birth has been previously reported in clinical studies, which indicate that fetal growth-restricted neonates can exhibit similar cardiac output relative to control groups even with a decrease in stroke volume, all due to a compensatory increase in heart rate.^49^ Similarly, we suggest that this increase in heart rate in our Δ9-THC offspring was to compensate for the decreased stroke volume in order to maintain stable levels of cardiac output to supply adequate blood to vital organs during development. However, at 3 weeks, after the hearts caught up in growth, we observed thicker LVAW accompanied by impaired cardiac function, including decreased stroke volume and cardiac output with preserved ejection fraction. Similar impairments of left ventricular function and hypertrophy have also been reported in IUGR models of maternal hypoxia.^50^ Given this and the trending (*p* = 0.1) rise in wall thickening for other regions and points of contraction (i.e., LVPW; diastole), we anticipate that cardiac hypertrophy may further progress with age as clearly demonstrated in hypoxic and nutrient models of fetal growth restriction;^50,62^ however, long-term studies need to be conducted. One common stressor that induces hypertrophic remodelling is hypertension, which can result in a region-specific (LVPW) hypertrophy.^63^ The trending increase in LVPW thickness *could* suggest that hypertension is playing a contributing role; however, 3 weeks might be too early given a similar model of IUGR (e.g., nicotine) exhibited increased cardiac posterior wall thickness at ~3 months.^62^ Moreover, in 4-month-old hypoxia-induced IUGR offspring, high blood pressure was not exhibited despite evidence of cardiac hypertrophy.^50^ Although still elusive, we suspect that the stressor is linked to postnatal catch-up growth exhibited in these Δ9-THC offspring, as previously demonstrated in other models of IUGR (e.g., nicotine).^61,62^ Given the cardiac output deficits observed and potential long-term cardiac hypertrophy, it is tempting to speculate that these offsprings could exhibit an early progression of diastolic dysfunction, which occurs in hypertrophic rat hearts of IUGR adult offspring,^50^ although more long-term studies are warranted. Ultimately, it is quite remarkable that we observe impaired cardiac function and decreased efficiency at pre-adolescence; therefore, it will be important to examine if this persists or rectifies in exposed offspring before the development of myocardial disease.

As the Δ9-THC offspring exhibited signs of ventricular dysfunction (e.g., decreased cardiac output) and ventricular hypertrophy (e.g., thicker LVAW), we next examined whether this is associated with deleterious characteristics of ventricular hypertrophy such as increased collagen deposition, which promotes fibrosis and stiffening.^64–66^ Fibrosis may be an important contributing factor to ventricular dysfunction and dilated cardiomyopathy.^66–68^ It is well established that increased collagen deposition is a characteristic of an aging heart.^51,69,70^ However, previous models of hypoxia-induced IUGR using rats have revealed significant collagen deposition (i.e., higher collagen I and III) in IUGR offspring early at 4 months.^50^ Strikingly, our animals exposed to Δ9-THC *in utero* exhibited higher transcript levels of collagen 3 as early as 3 weeks. Further, Western blot analysis reveals that protein levels of both collagen 1 and 3^69^ were significantly increased in the exposed group relative to the control group. This is interesting because this is earlier than expected, which leads us to suspect that the observed effects were exacerbated by postnatal catch-up growth. An increase in collagen content associated with catch-up growth has been demonstrated in maternal nicotine-exposed offspring.^62^ Although these models typically report fibrosis in adulthood, it is also important to note that earlier signs of fibrosis are apparent in a model of maternal hypoxia-induced IUGR whereby offspring exhibited increased collagen content and cross-linking structure as early as PND7.^71^ Given the early changes in extracellular collagen in the heart, long-term studies are warranted to examine if this could progress to cardiac stiffening and decreased contractility, as observed in other models of IUGR.^50^ In addition to increased protein expression of collagen, Δ9-THC-exposed offspring at 3 weeks also exhibited decreased cardiac protein expression of MMP-2. The MMPs are a group of collagenases that regulate collagen deposition, and downregulation of MMP-2 specifically has been attributed to disrupting collagen degradation in age-associated fibrosis in rat hearts.^72^ The reduction in MMP-2 protein expression observed in Δ9-THC-exposed IUGR offspring is consistent with previously reported decreases in hypoxia-induced IUGR rat offspring.^50^ In the hypoxia-induced model of IUGR, it was also found that ventricular relaxation was impaired at 4 months.^50^ Collectively, this suggests that changes in increased cardiac collagen content due to postnatal catch-up growth in these Δ9-THC offspring could lead to accelerated age-related collagen deposition, which could underlie the cardiac defects observed as early as 3 weeks and possibly progressing to impaired contractility and worsened cardiac function.

Along with increased collagen expression, 3-week-old offspring exposed to Δ9-THC also demonstrated greater inactivation of cardiac GSK-3β as indicated by increased phosphorylation of the serine 9A residue. Recently, there has been an emergence in the literature for the role of GSK-3β in fibrotic signalling.^73,74^ More specifically, in the heart, a previous study utilizing isolated cardiac fibroblasts and embryonic mice fibroblasts with deleted GSK-3β indicate a profibrotic myofibroblast phenotype.^75^ In addition to fibrosis, deletion of GSK-3β is also linked to cardiac hypertrophy in fetal mice.^76^ Finally, in an ex vivo study, they demonstrate, utilizing a protein kinase, that phosphorylation (inactivate) of GSK-3β is required for cardiomyocytes to undertake hypertrophy.^52^ This could be an interesting marker to further explore as these Δ9-THC offsprings age because it is involved with numerous intracellular signalling pathways implicated in a number of myocardial diseases.^53^

Our study has a few limitations and future directions. First, our study did not examine the effects on female offspring. However, it should be noted that at 3 weeks of age, rats are sexually immature, indicating that differences in sex steroids will unlikely contribute to any sex-specific cardiac effects. We have previously published that Δ9-THC-exposed female offspring do not exhibit differences in circulating estrogen and testosterone compared to control, but it remains plausible that there might be some underlying epigenetic differences at this early age.^46^ Another limitation of the study is that we did not examine how Δ9-THC might influence the great vessels of the heart. Third, while we assessed the effects of gestational exposure to Δ9-THC on postnatal cardiac dysfunction, future studies should also consider other developmental windows (pre-pregnancy, lactation, or both) of exposure. In addition, although we focused on postnatal outcomes, *in utero* analysis using Doppler velocimetry could help further characterize the potential *in utero* cardiac remodelling and type of FGR associated with changes in hemodynamic flow.^77–79^ For example, one clinical study demonstrated that in early-onset IUGR fetuses abnormal echocardiography and Doppler readings in the umbilical vein are associated with changes in cardiac morphology.^79^ Moreover, studies are also required to address if cannabidiol (the largest non-psychoactive component of *cannabis*) is safe for fetal and postnatal cardiovascular health. Finally, further work is warranted to examine if these Δ9-THC offsprings exhibit other indices of the metabolic syndrome given the expression of CB1R and CB2R in developing metabolic organs.^19–28^

In summary, this study demonstrates for the first time that prenatal exposure to Δ9-THC alone leads to cardiac dysfunction in postnatal life. Second, we identified some of the molecular cardiac targets underlying the early cardiac dysfunction in these gestational Δ9-THC-exposed offspring. Given the high rate of maternal cannabis consumption coupled with increased legalization in North America,^2,80^ understanding the long-term effects of *in utero* cannabinoid (e.g., Δ9-THC) exposure on postnatal cardiac health is of great importance. Moreover, the increase in Δ9-THC concentrations in cannabis over the past decade introduces the potential for more severe effects.^31^ The observed effects of Δ9-THC exposure on the offspring may be directly due to Δ9-THC (via CB1R and CB2R) impeding the fetal heart development or indirectly attributed to placental insufficiency and postnatal catch-up growth.^11,46^ We believe that direct effects are involved for a few reasons: (1) Δ9-THC is known to cross the placenta,^38^ (2) the cannabinoid receptors are expressed in the fetal heart, and (3) cannabinoid receptor agonists can directly impair cardiomyocyte growth in isolated neonatal rat cardiomyocytes.^28^ We argue that indirect effects are also involved given that exposure to Δ9-THC during gestation impairs placental sufficiency resulting in FGR,^11^ which is associated with cardiovascular disease later in life.^48,55,56^ Regardless, the outcomes of these direct and indirect effects of Δ9-THC pose serious safety concerns on long-term cardiac function in cannabinoid-exposed offspring. It is noteworthy that we have recently demonstrated that Δ9-THC offspring exhibit sex-specific dysglycemia,^46^ but the effects on lipid (i.e., cholesterol and triglyceride synthesis) homeostasis remain elusive. Collectively, these metabolic parameters could further impact cardiovascular deficits in these offspring later in life.
